# *Bacteroides* isolated from four mammalian hosts lack host-specific 16S rRNA gene phylogeny and carbon and nitrogen utilization patterns*

**DOI:** 10.1002/mbo3.159

**Published:** 2014-02-17

**Authors:** Todd Atherly, Cherie J Ziemer

**Affiliations:** USDA – Agricultural Research Service, National Laboratory for Agriculture and the EnvironmentAmes, Iowa, 50010-3120

**Keywords:** *Bacteroides*, cow, fecal bacteria, goat, gut microbiota, human, phenotype array, pig

## Abstract

One-hundred-and-three isolates of *Bacteroides ovatus*,*B. thetaiotaomicron*, and *B. xylanisolvens* were recovered from cow, goat, human, and pig fecal enrichments with cellulose or xylan/pectin. Isolates were compared using 16S rRNA gene sequencing, repetitive sequence-based polymerase chain reaction (rep-PCR), and phenotypic microarrays. Analysis of 16S rRNA gene sequences revealed high sequence identity in these *Bacteroides*; with distinct phylogenetic groupings by bacterial species but not host origin. Phenotypic microarray analysis demonstrated these *Bacteroides* shared the ability to utilize many of the same carbon substrates, without differences due to species or host origin, indicative of their broad carbohydrate fermentation abilities. Limited nitrogen substrates were utilized; in addition to ammonia, guanine, and xanthine, purine derivatives were utilized by most isolates followed by a few amino sugars. Only rep-PCR analysis demonstrated host-specific patterns, indicating that genomic changes due to coevolution with host did not occur by mutation in the 16S rRNA gene or by a gain or loss of carbohydrate utilization genes within these *Bacteroides*. This is the first report to indicate that host-associated genomic differences are outside of 16S rRNA gene and carbohydrate utilization genes and suggest conservation of specific bacterial species with the same functionality across mammalian hosts for this *Bacteroidetes* clade.

## Introduction

The gastrointestinal tracts (GIT) of vertebrates are among some of the most densely populated environments for microbes, with cell counts of ∼10^10^–10^12^ cells per mL (Ley et al. 2006; Thomas et al. 2011). These microbial populations play an important role in the development of the immune system, health, and nutrition of the vertebrate host (Dethlefsen et al. 2007). The advent of 16S rRNA gene sequence phylogeny has allowed for analysis of whole microbial communities. Intestinal bacterial populations show a low diversity at the phyla level, dominated by *Firmicutes* and *Bacteroidetes*; other phyla represented include *Protobacteria*,*Actinobacteria*,*Fusobacteria*,*Verrucomicrobia*,*Fibrobacteres*, and *Spyrochaeates* (Eckburg et al. 2006; Ley et al. 2008a). At the species and strain levels, the amount of diversity increases dramatically which has been partially attributed to host selective pressure and coevolution (Bäckhed et al. 2005; Ley et al. 2006).

The intestinal bacteria and their hosts live together in mutualistic relationships. The microbes that reside in the GIT have the capacity to produce enzymes that degrade dietary polysaccharides from plant cell walls that the host's enzymes cannot (Salyers 1984; Flint et al. 2008). The fermentation of these polysaccharides produces end products, such as short-chain fatty acids (SCFA), that the host uses as nutritional sources (Martens et al. 2011). The amount of energy supplied by the SCFA produced can be as much as 70% in ruminants, 20–30% in many omnivorous animals, and 10% in humans (Bergman 1990).

The intricate mutualistic relationship of the microbiota in the distal gut and the host results in an environment that allows for the coevolution of both microbiota and host. This coevolution, defined as the reciprocal adaptations of each lineage in response to the other, has resulted in differences in microbial composition among host species and is helpful in understanding the relationships between host and microbiota (Zaneveld et al. 2009). Comparison of microbial community 16S rRNA gene sequences from fecal samples across a wide range of host species revealed that microbial communities codiversified with their host (Ley et al. 2008b); suggesting evolutionary interaction between a host and their microbiota (Ley et al. 2008b). Coevolution could also be investigated using a single bacterial species or group that is found in multiple host species. These bacteria could be compared directly for shared ancestry; one would expect to find subpopulations specific to each host (Oh et al. 2010).

Eight week continuous culture enrichments of feces with cellulose and xylan/pectin (2/1) were used to isolate bacteria from the polysaccharide degrading microbial populations using feces from four hosts: cows (*n* = 4), goats (*n* = 4), humans (*n* = 4), and pigs (*n* = 6) (Ziemer 2013). A total of 1650 bacteria were isolated and identified using 16S rRNA gene sequencing; phyla distributions were similar across hosts; averaging 45.1% Firmicutes, 32.7% Bacteroidetes, 12.4% Proteobacteria, 2.6% Actinobacteria, 5.5% Fusobacteria, and 0.7% Synergistestes. These results are similar to those reported by Ley et al. (2008a) for feces, in 60 different mammalian species, Firmicutes and Bacteroidetes made up 82% of the nearly 20,000 classified sequences with 14.2% in the Proteobacteria, Fusobacteria, and Actinobacteria phyla. Nearly 75% (402 of 540) of Bacteroidetes isolates were identified as *B. ovatus, B. thetaiotaomicron,* or *B. xylanisolvens,* making this one of the predominant groups. Multiple isolates with slightly different 16S rRNA gene sequences were isolated from enrichments from each fecal donor.

Subgroups of *Bacteroides* species have been found within different hosts suggesting that they coevolved together (Bäckhed et al. 2005). The gastrointestinal *Bacteroides* are most often transmitted from parent to offspring (Ley et al. 2006). *Bacteroides* species are anaerobic, nonmotile, gram-negative rods and are one of the most numerous bacteria found in the colon of many different animal species (Thomas et al. 2011). *Bacteroides* have an unprecedented ability to degrade both plant polysaccharides and host mucins. A predominant group of *Bacteroides* species, including *B. ovatus*,*B. thetaiotaomicron*, and *B. xylanisolvens*, from multiple hosts (pig, cow, goat, and human) were characterized using 16S rRNA gene phylogenetic analysis, repetitive sequence-based PCR (rep-PCR) genomic banding patterns, and phenotypic microarrays of carbon and nitrogen metabolism to determine if differences due to host origin could be detected.

## Experimental Procedures

### Isolations

*Bacteroides* isolates were obtained from enrichments of fecal samples taken from four cows, four humans, four goats, and six pigs. Freshly voided feces (at least 500 g) were collected from individuals and returned to the lab for processing within 30 min. Continuous culture enrichments with cellulose and xylan–pectin mixture were performed for 8 weeks with each fecal sample as described in Ziemer (2013). Host type is designated in the isolate names as follows: NLAE-zl-Cx for isolates from cow, NLAE-zl-Gx for isolates from goat, NLAE-zl-Px for isolates from pig or NLAE-zl-Hx for isolates from human fecal enrichment cultures (see Table S1 for NCBI Accession Numbers). The isolates were fairly evenly distributed across individuals averaging five per cow, eight per goat, seven per human, and six per pig. Type strains, *B. ovatus* ATCC 8483 (X83952) (ATCC, Manassas, VA), *B. thetaiotaomicron* VPI 5482 (L16489) (ATCC), and *B. xylanisolvens* XB1A (AM230650) (DSMZ, Braunschweig, Germany) were included in analyses. A total of 85 cow, 126 goat, 77 human, and 114 pig *Bacteroides*, within *B. ovatus, B. thetaiotaomicron,* and *B. xylanisolvens* species clade were isolated in pure culture and DNA was extracted using DNeasy kit (QIAGEN Sciences, Germantown, MD). These isolates were analyzed by 16S rRNA gene sequencing and repetitive sequence-based PCR (rep-PCR) as described below. All of the 16S rRNA gene sequences were within 95% similarity regardless of *Bacteroides* species with no host-specific clustering. Isolates with rep-PCR banding patterns that were unique within a *Bacteroides* species were selected for further analysis. When rep-PCR patterns were identical among isolates within a *Bacteroides* species from a single fecal donor, the isolates were considered clonal and one isolate was selected as representative. When isolates from the same host type but different fecal donors demonstrated similar rep-PCR patterns then one isolate representing each fecal donor was chosen. The final number of isolates for analysis was 103 total: 17 *B. ovatus*, 32 *B. thetaiotaomicron*, and 54 *B. xylanisolvens* (21 cow, 31 goat, 29 human, and 22 pig isolates).

### 16S rRNA gene sequencing

16S rRNA gene sequencing of isolates used the 27f and 1492r primers (Lane 1991) in a 50 *μ*L reaction. The reactions contained 5 *μ*L 10× PCR buffer with 15 mmol/L MgCl_2_, 0.5 *μ*L of 10 mmol/L dNTPs, 1 *μ*L each of 25 *p*mol/L primer, 0.125 *μ*L of 5 U/*μ*L Taq polymerase (Invitrogen, Carlsbad, CA), 40 *μ*L of nuclease-free water, and ∼100 ng of sample DNA. A MJ Research PTC-220 thermocycler (MJ Research, St. Bruno, Quebec, Canada) was used with the following program: 95°C for 5 min and then for 30 cycles at 95°C for 45 sec, 47°C for 1 min, 72°C for 1 min, followed by a 72°C for 7 min final extension. Sequencing was done on a 3730xl DNA Analyzer (Applied Biosystems, Carlsbad, CA) by the Iowa State University DNA Sequencing and Synthesis Facility (Ames, IA).

Consensus sequences were generated using VNTI 11.1 software (Invitrogen), and those ≥1200 bp in length were analyzed using Ribosomal Database Project II (Cole et al. 2009) Sequence Match for identification. All sequences have been deposited in GenBank (Accession numbers presented Table S1). For this experiment, bacteria that identified at ≥90% *B. ovatus*,*B. thetaiotaomicron*, and *B. xylanisolvens* were selected for further investigation. Sequences identified with *Bacteroides* strain designations were also included if homologous to the targeted clade. Sequence similarity was analyzed using Bionumerics (v. 6.5 software, Applied Maths, Austin, TX) and a dendogram created using standard pairwise alignment and unweighted pair group method with arithmetic mean (UPGMA) clustering.

### Repetitive sequence-based PCR

The bacterial isolates within the *B. ovatus – B. thetaiotaomicron – B. xylanisolvens* clade were analyzed using rep-PCR with BOXA1R primer and ERIC1R and ERIC2 primers (Versalovic et al. 1991). The rep-PCR reactions for the BOX primer contained 3.35 *μ*L 10× PCR buffer with 15 mmol/L MgCl_2_, 0.5 *μ*L of 10 mmol/L dNTPs, 1 *μ*L of 25 *p*mol/L BOXA1R primer, 0.125 *μ*L 5 U/*μ*L Taq polymerase (Invitrogen), and ∼100 ng of sample DNA in 25 *μ*L volume. The reactions for ERIC1R and ERIC2 were the same except that 5 *μ*L 10× PCR buffer with 15 mmol/L MgCl_2_ and 1 *μ*L each of 25 *p*mol/L ERIC1R and ERIC 2 primers, were used per 25 *μ*L reaction. All reactions were run on an MJ Research PTC-220 thermocycler (MJ Research). The BOX and ERIC reaction conditions were 95°C for 5 min and then for 30 cycles at 95°C for 45 sec, 50°C and 46°C, respectively, for 1 min, 72°C for 1 min, followed by 72°C for 7 min final extension. The PCR amplicons were separated by electrophoresis on a 1.5% Agarose (Amresco, Solon, OH) gel in 1× TBE buffer for 6 h at 100 V with 100 bp molecular weight marker (Amresco). Gels were stained with ethidium bromide and pictured under ultraviolet light using the Kodak Image Station 4000MM Pro (Molecular Imaging Systems, Carestream Health Inc., Rochester, NY). Analysis of the gel banding patterns was done using BioNumerics (Applied Maths) and dendograms were generated using Pearson correlation similarity coefficient with optimization of 1% and the UPGMA clustering method.

### Phenotypic comparison

The 103 isolates were then screened for their ability to metabolize 190 carbon and 95 nitrogen sources using phenotypic microarrays (PM1 and PM2A for carbon and PM3B for nitrogen) (Biolog, Hayward, CA), according to manufacturer's instructions without modifications (Mapp-ley et al. 2012). Microarray plates were inoculated and incubated anaerobically for 24 h then read using a Biolog MicroStation reader. Growth was measured using an optical density at 590 nm for PM1 and PM2A an optical density at 750 nm for PM3B. The following carbon sources were not analyzed due to abiotic reactions under anaerobic experimental conditions: arabinose, dihyderoxyacetone, D-gluosamine, 2-hydroxybonzoic acid, 5-keto-D-gluconate, L-lyxose, palatinose, D-ribose, 3-deoxy-D-ribose, sorbic acid, D-tagatose, and D-xylose (Borglin et al. 2009; Mappley et al. 2012). The values were zeroed to account for background noise and any results ≤0.1 O.D. (>2 times background O.D.) were considered to have no growth. In order to assess repeatability of the phenotypic arrays a subset of isolates were analyzed on two to five plates. The two carbon utilization arrays were combined for analysis of utilization patterns. Analysis of the data was done using BioNumerics (Applied Maths) for carbon and nitrogen substrate sets. The dendograms were generated using the Pearson correlation similarity coefficient and UPGMA clustering method.

## Results

### 16S rRNA phylogeny

**Figure 1:**
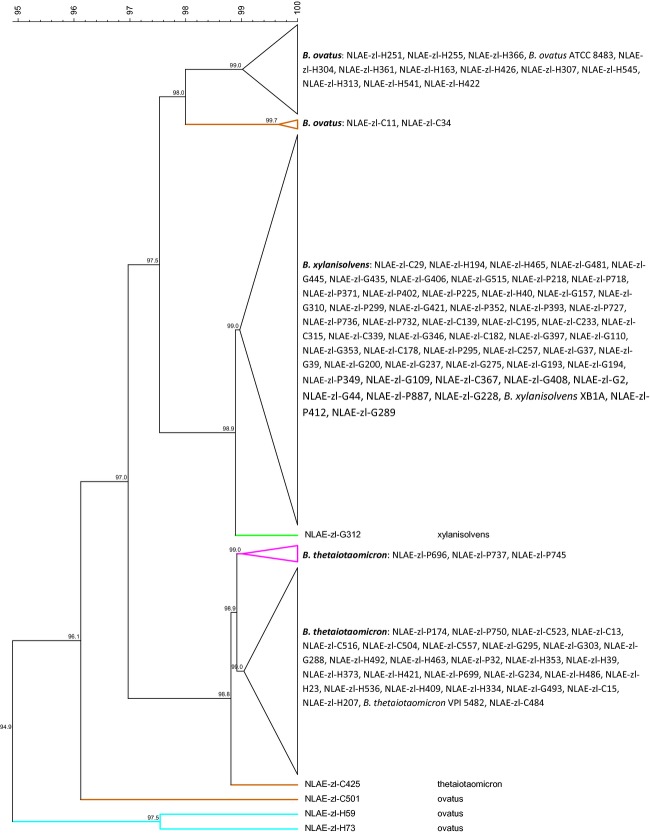
Cluster analysis of 16S rRNA genes using UPGMA, dendogram with similarity values (%). Bar at top is similarity continuum. Differentiation among host by isolate name: cow = NLAE-zl-Cx, goat = NLAE-zl-Gx, human = NLAE-zl-Hx, and pig = NLAE-zl-Px.

Analysis of 16S rRNA gene sequences for *B. thetaiotaomicron*,*B*. *ovatus*, and *B*. *xylanisolvens* isolates and type strains (ATCC, Manassas, VA; DSMZ, Braunschweig, Germany) demonstrate all were within ≥97% similarity to each other (Fig. 1). There were three exceptions, all *B. ovatus* strains, one cow isolate (NLAE-zl-C501) at 96.1%, and two human isolates (NLAE-zl-H59 and NLAE-zl-H73) at 94.9% similarity with the other isolates. The isolates formed distinct phylogenetic groupings by bacterial species with 17 isolates grouped with *B*. *ovatus*, 32 with *B*. *thetaiotaomicron*, and the remaining 54 with *B*. *xylanisolvens* type strains. *Bacteroides ovatus* strains were isolated from human (14) and cow (3) fecal enrichments. These isolates distinctly grouped by host origin with 98% sequence similarity. The *B*. *thetaiotaomicron* isolates (seven pig, eight cow, five goat, and 12 human) had ≥98.6% sequence similarity with no differentiation due to host origin. Similarly, the *B*. *xylanisolvens* isolates (15 pig, 10 cow, 26 goat, and three human) had ≥98.9% sequence similarity, again, no effects of host origin.

**Figure 2:**
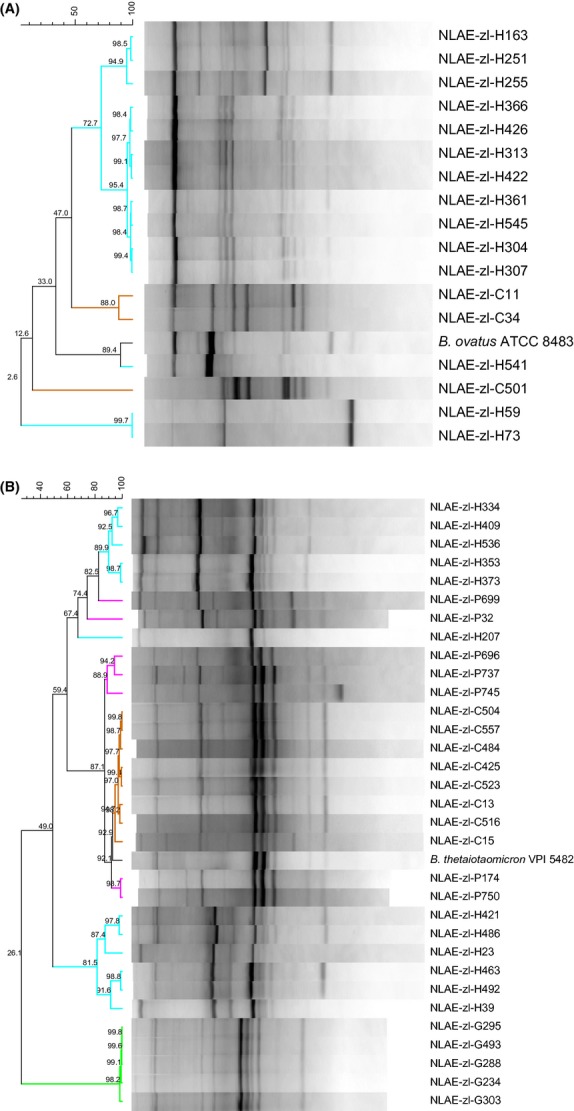
Cluster analysis of BOX primer rep-PCR. Similarity values (%). (A) *Bacteroides ovatus*; (B) *B. thetaiotaomicron*. Differentiation among host by isolate name: cow = NLAE-zl-Cx, goat = NLAE-zl-Gx, human = NLAE-zl-Hx, and pig = NLAE-zl-Px.

**Figure 3:**
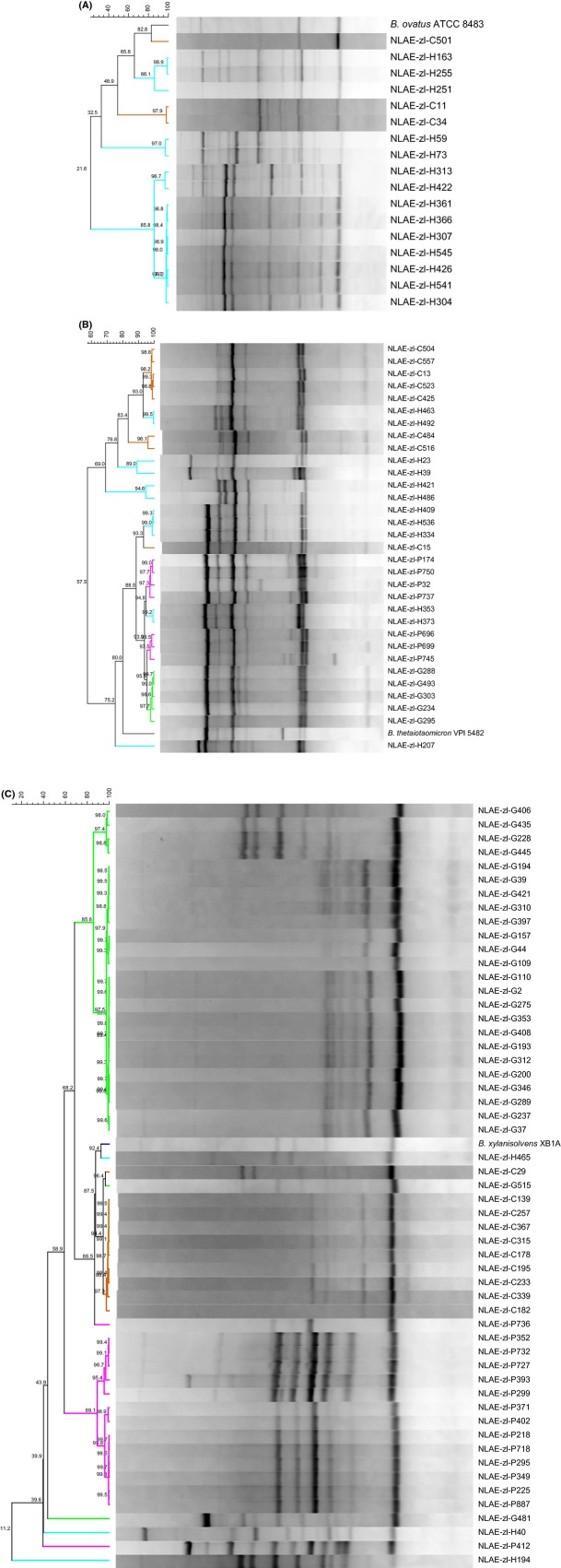
Cluster analysis of ERIC primers rep-PCR. Similarity values (%). (A) *Bacteroides ovatus*; (B) *B. thetaiotaomicron*; (C) *B. xylanisolvens*. Bar at top is similarity continuum. Differentiation among host by isolate name: cow = NLAE-zl-Cx, goat = NLAE-zl-Gx, human = NLAE-zl-Hx, and pig = NLAE-zl-Px.

Analysis of the rep-PCR banding demonstrated distinct patterns with both the BOX and ERIC primers by *Bacteroides* species and host origin, results are presented by *Bacteroides* species (Figs. 2, 3). Within *B. ovatus,* isolate banding patterns from BOX rep-PCR formed three groups of human isolates with ≥90% similarity except for isolate NLAE-zl-H541 which had 89.4% similarity with the type strain (*B. ovatus* ATCC 8483) (Fig. 2A). The BOX rep-PCR banding patterns of the two human (NLAE-zl-H59 and NLAE-zl-H73) and one cow (NLAE-zl-C501) isolates with less than 97% 16S rRNA gene sequence similarity were distinct from the other *B. ovatus* isolates. The ERIC rep-PCR produced four groups of *B. ovatus* human isolates with ≥90% similarity (Fig. 3A). The banding patterns of NLAE-zl-H59, NLAE-zl-H73, and NLAE-zl-C501 were more similar to the rest of the isolates using the ERIC primers in contrast to results using BOX rep-PCR. In fact the cow isolate, NLAE-zl-C501, has the highest banding pattern similarity to the type strain *B. ovatus* ATCC 8483 at 82.8%.

Analysis of banding patterns generated using the BOX rep-PCR for *B. thetaiotaomicron* isolates at ≥90% similarity formed one group of goat isolates, four groups of human isolates, one group of pig isolates, and one group of a mix of pig and cow isolates. Within this mixed group, the type strain *B. thetaiotaomicron* VPI 5482 is also found (Fig. 2B). The ERIC primers produced one cow, three human, and three mixed groups with ≥90%. Two of the mixed groups contain human and cow isolates, where the third group contains goat, human, and pig isolates. The *B. thetaiotaomicron* VPI 5482 type strain and NLAE-zl-H207 did not group with any of the other isolates (Fig. 3B).

Repetitive sequence-based PCR of *B. xylanisolvens* using the BOX primer resulted in very few bands, ranging in number between 1 and 4, thus was not used to assess *B. xylanisolvens* genetic diversity. Using a ≥90% similarity cutoff, ERIC rep-PCR banding pattern analysis resulted in two goat, two pig, and one mixed, cow and goat, isolate groupings. The type strain *B. xylanisolvens* XB1A was most similar to a human isolate, NLAE-zl-H465, at 91.9%. Four isolates (NLAE-zl-H194, NLAE-zl-H40, NLAE-zl-P412, and NLAE-zl-G481) had distinct banding patterns compared to the other *B. xylanisolvens* isolates (Fig. 3C).

### Substrate utilization

**Figure 4:**
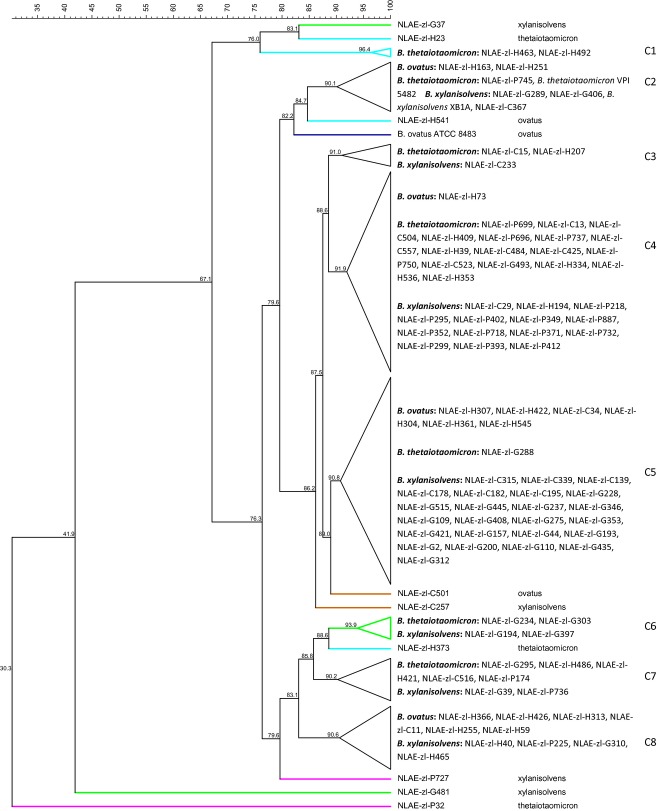
Cluster analysis of carbon substrate plates PM1 and PM2A utilization. Similarity values (%). Bar at top is similarity continuum. Differentiation among host by isolate name: cow = NLAE-zl-Cx, goat = NLAE-zl-Gx, human = NLAE-zl-Hx, and pig = NLAE-zl-Px.

Analysis of isolate carbon and nitrogen utilization patterns produced with the phenotypic microarrays did not demonstrate distinct utilization patterns by *Bacteroides* species or host. The carbon utilization patterns had 76.3% similarity across 190 carbon substrates, for all but six isolates (Fig. 4). At ≥90% similarity level, there were eight carbon utilization pattern groupings. Groups C2, C4, and C5 contained all three *Bacteroides* species from at least three hosts, groups C3, C6, and C7 contained *B. thetaiotaomicron* and *B. xylanisolvens* from at least two hosts, group C8 contained *B. ovatus* and *B. xylanisolvens* from all four hosts and finally grouping C1 contained *B thetaiotaomicron* from human enrichments. Groups C4 and C5 contained the most isolates with 31 in each (C4: 1, 16, and 14 and C5: 6, 1, and 24 numbers of *B. ovatus, B. thetaiotaomicron,* and *B. xylanisolvens*, respectively) The type strains *B. thetaiotaomicron* VPI 5482 and *B. xylanisolvens* XB1A are found in the same group (C2) and had 93.7% similarity for carbon utilization patterns. The type strain *B. ovatus* ATCC 8483 did not group with any other isolates but was 82.2% similar to the other type strains. Two isolates (NLAE-zl-G481 and NLAE-zl-P32) have low percent similarity to the other isolates, 41.9% and 30.3%, respectively, due to positives across all 95 carbon substrate on a plate; NLAE-zl-G481 on plate PM2 and NLAE-zl-P32 on plate PM1. All of the *Bacteroides* isolates were able to utilize 11 carbon substrates: galactose, mannose, xylose, psicose, lyxose, allose, arabinose, 2-deoxy-D-ribose, 3-methylglucose, tagatose, and 5-keto-D-gluconic acid (Tables S2, S3). *Bacteriodes ovatus* isolates had the broadest range of carbon substrate utilization with an additional 20 substrates utilized by all strains: fructose, *α*-D-glucose, maltose, melibiose, *α*-D-lactose, lactulose, sucrose, D-fructose-6-phosphate, maltotriose, glucuronamide, n-acetyl-D-galactosamine, 3-O-*β*-D-galactopyranosyl-D-arabinose, and *β*-methyl-D-galactoside. *Bacteriodes thetaiotaomicron* and *B. xylanisolvens* were all able to utilize three (rhamnose, maltotriose, and glucosamine) and four (rhamnose, fructose, a-D-glucose, and glucuronamide) additional carbon substrates, respectively.

**Figure 5:**
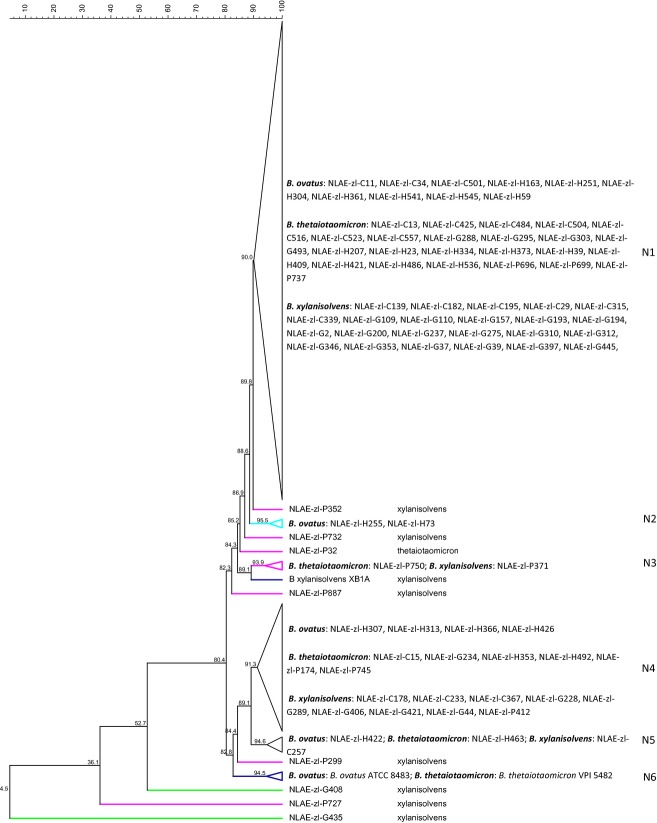
Cluster analysis of nitrogen substrate plate PM3B utilization. Similarity values (%). Bar at top is similarity continuum. Differentiation among host by isolate name: cow = NLAE-zl-Cx, goat = NLAE-zl-Gx, human = NLAE-zl-Hx, and pig = NLAE-zl-Px.

Using ≥90% similarity to assess nitrogen utilization patterns across 95 nitrogen substrates resulted in six groupings (Fig. 5). All three *Bacteroides* species from the four hosts were found in N1, N4, and N5 groupings, group N3 contained *B. thetaiotaomicron* and *B. xylanisolvens* from pigs, N2 contained *B. ovatus* from humans and N6 contained the *B. ovatus* and *B. thetaiotaomicron* type strains at 94.5% utilization similarity. Group N1 contained the largest number of isolates with 10 *B. ovatus*, 23 *B. thetaiotaomicron*, and 23 *B. xylanisolvens*. Isolate NLAE-zl-G435 had an extremely low similarity to the others, 4.5%, due to positives across all 95 nitrogen substrates. There were no nitrogen substrates that could be utilized by all of these *Bacteroides* isolates (Table S4). The nitrogen substrates most utilized were the purine derivatives xanthine, a nucleobase, and guanine. All *B. ovatus and B. thetaiotaomicron* isolates utilized xanthine, all *B. ovatus* isolates also utilized guanine; however, there were no nitrogen substrates that were utilized by all *B. xylanisolvens* isolates. The amino sugars D-galactosamine and D-mannosamine (*B*. *ovatus* 5 and 6, *B. thetaiotaomicron* 9 and 12, *B. xylanisolvens* 15 and 14 isolates, respectively), N-acetyl-D-glucosamine and N-acetyl-D-galactosamine (*B*. *ovatus* 5 and 5, *B. thetaiotaomicron* 14 and 10, *B. xylanisolvens* 19 and 21 isolates, respectively), as well as the amino acids L-homoserine and L-ornithine (*B*. *ovatus* 4 and 4, *B. thetaiotaomicron* 9 and 7, *B. xylanisolvens* 12 and 10 isolates, respectively), were used by a moderate number of our isolates.

## Discussion

Most of the research involving *Bacteroides* species has been done on isolates taken from humans (Salyers et al. 1977; Salyers 1984; Xu et al. 2007; Zocco et al. 2007; Martens et al. 2009). In this study, we wanted to compare *Bacteroides* isolates within the *B. thetaiotaomicron–ovatus–xylanisolvens* clade obtained from different mammalian hosts. Our isolation of 103 bacteria within this clade demonstrated their presence in the GIT of mammals with varying diets and digestive physiologies. However, the *Bacteroides* species in this clade were not equally distributed among host species. *Bacteroides ovatus* was recovered primarily in human and cow fecal enrichments. The *B. xylanisolvens* were isolated from all host fecal enrichments, but they were the most abundant from the goat fecal enrichments and least abundant from the human fecal enrichments. The *B. thetaiotaomicron* was also isolated from all host fecal enrichments with greatest abundance in isolates from pig fecal enrichments and relatively even distribution among other hosts. The primary host difference was more *B. ovatus* and *B. thetaiotaomicron* and less *B. xylanisolvens* isolates were from humans while in isolates from cow, goat, and pig *B. xylanisolvens* isolates predominated.

Host-specific differences were expected for rep-PCR patterns as well as carbon and nitrogen substrate utilization. Based on analysis of fecal microbiota in 60 mammals using community 16S rRNA gene sequences indicating the influence of host diet type and digestive physiology (Ley et al. 2008a,b2008b), there was potential to see host-specific or ruminant verses nonruminant patterns in the 16S rRNA gene sequences of our *Bacteroides* isolates. Only rep-PCR patterns supported genomic differences among *B. ovatus, B. thetaiotaomicron,* and *B. xylanisolvens* isolates due to host origin, indicative of coevolution in intestinal bacteria with their host. Neither the 16S rRNA gene sequences nor carbon and nitrogen substrate utilization were affected by the host they were isolated from. That 16S rRNA gene sequences do not reflect differences in host origin is not surprising due to its inherent properties: universal distribution and function as well as high sequence conservation (Woese 2006). The specific environment and other members of the resident microbial community would influence mutations and horizontal gene transfer that could lead to genomic differences across strains of the same bacterial species. The rep-PCR data demonstrate host-specific patterns within the genomes of the *B. ovatus-thetaiotaomicron-xylanisolvens* clade but the phenotypic array data suggest that the differences are not present in the carbohydrate utilization genes or if differences are there they maintain functional fidelity.

While phylogentic grouping due to host origin was evident for *B. ovatus* isolates, there was no host origin distinction among *B. thetaiotaomicron* and *B. xylanisolvens* 16S rRNA gene sequences. The *B. thetaiotaomicron* and *B*. *xylanisolvens* were obtained from all of the host fecal enrichments, while *B. ovatus* was obtained only from human and cow. *Bacteroides ovatus* isolates from cow and human had 2.0–2.8% difference in 16S rRNA gene sequence similarity, approaching the difference to separate bacterial species. The two human *B. ovatus* isolates, NLAE-zl-H59 and NLAE-zl-H73 were only 96.5% similarity to the other *B. ovatus* and may represent a different *Bacteroides* species. In the context of this study, the 16S rRNA gene sequence was not able to determine the host origin of *B. thetaiotaomicron* and *B. xylanisolvans* isolates.

The rep-PCR method targets repetitive DNA sequences that are interspersed in the genome of the organism (Versalovic et al. 1994) and is used to visualize difference in the genomes. The amplification of these products can then be used to differentiate among strains of bacterial species. The *Bacteroides* are known to have mobile genetic elements such as conjugative transposons, plasmids, and mobilizable transposons (Salyers 1984; Wexler 2007). These elements can allow for the uptake of foreign DNA from other microbes in the environment and possibly from the host (Comstock and Coyne 2003). On the basis of this information and reports of microbial communities across multiple host types (Dethlefsen et al. 2007; Ley et al. 2008a), we anticipated differences in rep-PCR banding patterns due to mobilizable genetic elements and host origin. Repetitive sequence-based PCR banding patterns grouped *B. ovatus* and *B. xylanisolvens* isolates by host origin. The *B. thetaiotaomicron* banding patterns resulted in groups of isolates from single and multiple host origin. The evidence of rep-PCR banding patterns grouping by host origin gives credence to a coevolution of the bacteria and host theory for *B. ovatus* and *B. xylanisolvens*, although less so for *B. thetaiotaomicron*.

Phenotypically, isolates from the three *Bacteroides* species shared the ability to utilize a number of the same carbon substrates, a reflection of their broad carbohydrate utilization. These results demonstrate functional redundancy; a predominant feature of the GIT microbiota (Ley et al. 2006), which would benefit the host by maintaining functionality of the intestinal microbiota even with the loss of a microbial lineage. This functional redundancy is considered to be host-driven selection (Ley et al. 2006), however, within the *B. thetaiotaomicron–ovatus–xylanisolvens* clade, host origin did not account for specific carbon utilization patterns. We speculate that the carbohydrate utilization functions of these *Bacteroides* are so important for utilization of plant cell wall carbohydrates that they are conserved across all mammalian intestinal microbiota. Taken together with the rep-PCR data, we also propose that the host-specific differences in the genomes of these *Bacteroides* species primarily come from genes not involved in carbohydrate utilization or any differences in carbohydrate utilization genes maintain precise functionality.

Our *Bacteroides* isolates showed an inability to utilize most nitrogen substrates on the nitrogen phenotypic array. Our results are supported by earlier findings that *B. fragilis* were unable to utilize free amino acids, peptides, nitrate, or urea as a nitrogen source (Varel and Bryant 1974). The lack of ammonia utilization by these isolates on the Biolog nitrogen phenotypic array was unexpected; thus, we used the media of Varel and Bryant (1974) to verify the results. Initial results demonstrated that the isolates could grow on the ammonia containing medium but also on the medium without ammonia; they were able to utilize the cysteine in the medium reducing agent (cysteine-HCL). This indicates that for results in the Varel and Bryant (1974) nitrogen source are confounded with the cysteine in the reducing agent. We then repeated the test using sodium-thioglycolate (Sigma-Aldrich) as the reducing agent which confirmed that isolates could utilize ammonia and cysteine. This makes two nitrogen substrates that gave false negatives on the Biolog nitrogen phenotypic array. While we did not have concerns about false-positive reactions, there were no abiotic reactions for any nitrogen substrates, we utilized the sodium-thioglycolate-modified medium of Varel and Bryant (1974) to examine growth on animo sugars and purine derivatives using a subset of isolates and the type strains. The isolates were able to grow on D-mannosamine hydrochloride, N-acetyl-D-glucosamine, N-acetyl-D-glactosamine, and xanthine, guanine but failed to grow on L-ornithine monohydrochloride (a potential false positive on the Biolog plate). This supports the results of utilization of purine derivatives and amino sugars for some of the bacteria in the *B. thetaiotaomicron–ovatus–xylanisolvens* clade. Macfarlane and Gibson (1991) reported that *B. fragilis* was able to grow on the glycoprotein mucin as well as mucin components such as N-acetylglucosamine and N-acetylgalactosamine. We have sequenced the genomes of 24 isolates and they all contained one copy of glutamine synthase and 2–3 copies of glutamate dehydrogenase (T. Atherly and C. J. Ziemer, unpubl. data), which appear to be functional based on our in vitro utilization assays with the amino sugars and purine derivatives. These results indicate that the Biolog PM3 nitrogen phenotypic array is not a suitable method for determining nitrogen utilization of bacteria under anaerobic conditions. Nitrogen utilization in the *Bacteroides* has not been well-defined, the Varel and Bryant (1974) paper is the primary source, and most research has focused on *B. fragilis*. A more detailed analysis of nitrogen substrate utilization by the *Bacteroides*, using an alternative reducing agent to cysteine-HCL, is warranted.

Isolates from the *B. thetaiotaomicron–ovatus–xylanisolvens* clade were found in fecal enrichments from mammals of differing digestive physiology, both ruminant and monogastric. Specificity of a particular *Bacteroides* species to one host, gut physiology or diet was not seen. The rep-PCR analysis indicated that the isolates' genomes had been altered by the host environment to some degree but genomic differences do not appear to be in 16S rRNA or carbohydrate utilization genes. These *Bacteroides* isolates were able to utilize a wide range of the carbon substrates but very few of the nitrogen substrates examined. Plant cell wall carbohydrates, recalcitrant to mammalian digestive enzymes, are the primary carbon substrates in the hind gut of both ruminants and monogastrics. The lack of host-specific carbon and nitrogen utilization in our *Bacteroides* isolates indicates conservation of functionality across hosts. This is the first report to indicate that host-associated genomic differences are outside of 16S rRNA and carbohydrate utilization genes and to suggest conservation of specific bacterial species with the same functionality across mammalian hosts for this *Bacteroidetes* clade.

## Supporting Information

**Table S1.***Bacteroides* isolates with NCBI Accession Numbers by host origin and *Bacteroides* species. **Table S2.**List of phenotypic microarray substrates and the number of isolates by *Bacteroides* species that utilized the substrates of Biolog PM1. **Table S3.**List of phenotypic microarray substrates and the number of isolates by *Bacteroides* species that utilized the substrates of Biolog PM2A. **Table S4.**List of phenotypic microarray substrates and the number of isolates by *Bacteroides*species that utilized the substrates of Biolog PM3B.
