# Supplementary material for: Bacteroides isolated from four mammalian hosts lack host-specific 16S rRNA gene phylogeny and carbon and nitrogen utilization patterns
Source: Microbiologyopen. 2014 Feb 17;3(2):225–38. doi: 10.1002/mbo3.159 (PMC3996570; doi:10.1002/mbo3.159)
Supplement: Supplementary file 1 — Table S1.Bacteroides isolates with NCBI Accession Numbers by host origin and Bacteroides species. Table S2.List of phenotypic microarray substrates and the number of isolates by Bacteroides species that utilized the substrates of Biolog PM1. Table S3.List of phenotypic microarray substrates and the number of isolates by Bacteroides species that utilized the substrates of Biolog PM2A. Table S4.List of phenotypic microarray substrates and the number of isolates by Bacteroidesspecies that utilized the substrates of Biolog PM3B. [file mbo30003-0225-sd1.docx]

Supplemental Material

Supplemental Table 1. *Bacteroides* isolates with NCBI Accession Numbers by host origin and *Bacteroides* species.

| **Host** | ***Bacteroides ovatus*** | | ***Bacteroides thetaiotaomicron*** | | ***Bacteroides Xylanisolvens*** | |
| --- | --- | --- | --- | --- | --- | --- |
|  | **Isolate name** | **Accession #^a^** | **Isolate name** | **Accession #** | **Isolate name** | **Accession #** |
| Cow | NLAE-zl-C11 | JQ607822 | NLAE-zl-C13 | JQ607748 | NLAE-zl-C139 | JQ607878 |
|  | NLAE-zl-C34 | JQ607816 | NLAE-zl-C15 | JQ607764 | NLAE-zl-C178 | JQ607910 |
|  | NLAE-zl-C501 | JQ608202 | NLAE-zl-C425 | JQ608129 | NLAE-zl-C182 | JQ607913 |
|  |  |  | NLAE-zl-C484 | JQ608185 | NLAE-zl-C195 | JQ607923 |
|  |  |  | NLAE-zl-C504 | JQ608205 | NLAE-zl-C233 | JQ607957 |
|  |  |  | NLAE-zl-C516 | JQ608216 | NLAE-zl-C257 | JQ607975 |
|  |  |  | NLAE-zl-C523 | JQ608223 | NLAE-zl-C29 | JQ607779 |
|  |  |  | NLAE-zl-C557 | JQ608257 | NLAE-zl-C315 | JQ608029 |
|  |  |  |  |  | NLAE-zl-C339 | JQ608051 |
|  |  |  |  |  | NLAE-zl-C367 | JQ608078 |
| Goat |  |  | NLAE-zl-G234 | JX048267 | NLAE-zl-G109 | JX048146 |
|  |  |  | NLAE-zl-G288 | JX048308 | NLAE-zl-G110 | JX048541 |
|  |  |  | NLAE-zl-G295 | JX048567 | NLAE-zl-G157 | JX048149 |
|  |  |  | NLAE-zl-G303 | JX048570 | NLAE-zl-G193 | JX048593 |
|  |  |  | NLAE-zl-G493 | JX048479 | NLAE-zl-G194 | JX048237 |
|  |  |  |  |  | NLAE-zl-G2 | JX048075 |
|  |  |  |  |  | NLAE-zl-G20 | JX048535 |
|  |  |  |  |  | NLAE-zl-G200 | JX048277 |
|  |  |  |  |  | NLAE-zl-G228 | JX048302 |
|  |  |  |  |  | NLAE-zl-G237 | JX048284 |
|  |  |  |  |  | NLAE-zl-G275 | JX048306 |
|  |  |  |  |  | NLAE-zl-G289 | JX048315 |
|  |  |  |  |  | NLAE-zl-G310 | JX048573 |
|  |  |  |  |  | NLAE-zl-G312 | JX048309 |
|  |  |  |  |  | NLAE-zl-G346 | JX048368 |
|  |  |  |  |  | NLAE-zl-G353 | JX048344 |
|  |  |  |  |  | NLAE-zl-G37 | JX048070 |
|  |  |  |  |  | NLAE-zl-G39 | JX048086 |
|  |  |  |  |  | NLAE-zl-G397 | JX048391 |
|  |  |  |  |  | NLAE-zl-G406 | JX048400 |
|  |  |  |  |  | NLAE-zl-G408 | JX048401 |
|  |  |  |  |  | NLAE-zl-G421 | JX048413 |
|  |  |  |  |  | NLAE-zl-G435 | JX048426 |
|  |  |  |  |  | NLAE-zl-G44 | JX048116 |
|  |  |  |  |  | NLAE-zl-G445 | JX048435 |
|  |  |  |  |  | NLAE-zl-G481 | JX048468 |
|  |  |  |  |  | NLAE-zl-G515 | JX048501 |
| Human | NLAE-zl-H163 | JX006391 | NLAE-zl-H207 | JX006430 | NLAE-zl-H194 | JX006767 |
|  | NLAE-zl-H251 | JX006471 | NLAE-zl-H23 | JX006274 | NLAE-zl-H40 | JX006288 |
|  | NLAE-zl-H255 | JX006475 | NLAE-zl-H334 | JX006549 | NLAE-zl-H465 | JX006676 |
|  | NLAE-zl-H304 | JX006520 | NLAE-zl-H353 | JX006567 |  |  |
|  | NLAE-zl-H307 | JX006523 | NLAE-zl-H373 | JX006585 |  |  |
|  | NLAE-zl-H313 | JX006778 | NLAE-zl-H39 | JX006287 |  |  |
|  | NLAE-zl-H361 | JX006574 | NLAE-zl-H409 | JX006621 |  |  |
|  | NLAE-zl-H366 | JX006579 | NLAE-zl-H421 | JX006633 |  |  |
|  | NLAE-zl-H422 | JX006634 | NLAE-zl-H463 | JX006674 |  |  |
|  | NLAE-zl-H426 | JX006638 | NLAE-zl-H486 | JX006697 |  |  |
|  | NLAE-zl-H541 | JX006744 | NLAE-zl-H492 | JX006701 |  |  |
|  | NLAE-zl-H545 | JX006748 | NLAE-zl-H536 | JX006739 |  |  |
|  | NLAE-zl-H59 | KC107215 |  |  |  |  |
|  | NLAE-zl-H73 | KC107216 |  |  |  |  |
| Pig |  |  | NLAE-zl-P174 | JQ606991 | NLAE-zl-P218 | JQ607049 |
|  |  |  | NLAE-zl-P32 | JQ606978 | NLAE-zl-P225 | JQ607047 |
|  |  |  | NLAE-zl-P696 | JQ607518 | NLAE-zl-P295 | JQ606947 |
|  |  |  | NLAE-zl-P699 | JQ607479 | NLAE-zl-P349 | JQ607074 |
|  |  |  | NLAE-zl-P737 | JQ607550 | NLAE-zl-P352 | JQ607091 |
|  |  |  | NLAE-zl-P745 | JQ607554 | NLAE-zl-P371 | JQ607087 |
|  |  |  | NLAE-zl-P750 | JQ607576 | NLAE-zl-P402 | JQ607138 |
|  |  |  |  |  | NLAE-zl-P718 | JQ607540 |
|  |  |  |  |  | NLAE-zl-P727 | JQ607564 |
|  |  |  |  |  | NLAE-zl-P732 | JQ607549 |
|  |  |  |  |  | NLAE-zl-P887 | JQ607697 |

^a^ Accession # = Accession number for 16S rRNA sequence deposited in GenBank, NCBI.

Supplemental Table 2. List of phenotypic microarray substrates and the number of isolates by *Bacteroides* species that utilized the substrates of Biolog PM1.

| **Carbon substrate** | ***B. ovatus*** | ***B. thetaiotaomicron*** | ***B. xylanisolvens*** |
| --- | --- | --- | --- |
|  | n = 17 | n = 29 | n = 57 |
|  | 3 C, 14 H^a^ | 8 C, 5 G, 12 H, 7 P | 10 C, 26 G, 3 H, 15 P |
| L-Arabinose^b^ | 17^c^ | 31^c^ | 54^c^ |
| N-Acetyl-D-glucosamine | 17^c^ | 27^c^ | 50^c^ |
| D-Saccharic acid | 0 | 1 | 0 |
| Succinic acid | 1 | 1 | 0 |
| D-Galactose | 17^c^ | 32^c^ | 54^c^ |
| L-Aspartic acid | 2^c^ | 1^c^ | 1 |
| L-Proline | 2 | 1 | 0 |
| D-Alanine | 0 | 0 | 0 |
| D-Trehalose | 10^c^ | 7^c^ | 29^c^ |
| D-Mannose | 17^c^ | 32^c^ | 54^c^ |
| Dulcitol | 0 | 0 | 2 |
| D-Serine | 0 | 0 | 1 |
| D-Sorbitol | 3 | 2 | 3 |
| Glycerol | 0 | 1 | 2 |
| L-Fucose | 12^c^ | 29^c^ | 50^c^ |
| D-Glucuronic acid | 13^c^ | 28^c^ | 53^c^ |
| D-Gluconic acid | 2^c^ | 0^c^ | 0^c^ |
| DL-α-Glycerol phosphate | 2^c^ | 0 | 0 |
| D-Xylose^b^ | 17^c^ | 32^c^ | 54^c^ |
| L-Lactic acid | 11^c^ | 11^c^ | 21^c^ |
| Formic acid | 0 | 0 | 1 |
| D-Mannitol | 3^c^ | 2 | 2 |
| L-Glutamic acid | 1^c^ | 2 | 3 |
| D-Glucose-6-phosphate | 6^c^ | 5^c^ | 18^c^ |
| D-Galactonic acid-γ-lactone | 0 | 0 | 3 |
| DL-Malic acid | 0 | 0 | 2 |
| D-Ribose^b^ | 17^c^ | 32^c^ | 53^c^ |
| Tween 20 | 0 | 0 | 0 |
| L-Rhamnose | 16^c^ | 32^c^ | 54^c^ |
| D-Fructose | 17^c^ | 29^c^ | 54^c^ |
| Acetic acid | 5^c^ | 3^c^ | 7^c^ |
| α-D-Glucose | 17^c^ | 31^c^ | 54^c^ |
| Maltose | 17^c^ | 31^c^ | 52^c^ |
| D-Melibiose | 17^c^ | 29^c^ | 53^c^ |
| Thymidine | 15^c^ | 25^c^ | 38^c^ |
| L-Asparagine | 1^c^ | 1^c^ | 1^c^ |
| D-Aspartic acid | 0 | 0 | 0 |
| D-Glucosaminic acid | 1 | 0 | 1^c^ |
| 1,2-Propanediol | 0^c^ | 1^c^ | 0^c^ |
| Tween 40 | 0^c^ | 0^c^ | 1^c^ |
| α-Ketoglutaric acid | 2^c^ | 0^c^ | 2^c^ |
| α-Ketobutyric acid | 9^c^ | 6^c^ | 17^c^ |
| α-Methyl-D-galactoside | 6^c^ | 3^c^ | 9^c^ |
| α-D-Lactose | 17^c^ | 28^c^ | 52^c^ |
| Lactulose | 17^c^ | 26^c^ | 50^c^ |
| Sucrose | 17^c^ | 30^c^ | 52^c^ |
| Uridine | 17^c^ | 23^c^ | 46^c^ |
| L-Glutamine | 1^c^ | 2 | 2^c^ |
| m-Tartaric acid | 0 | 0 | 1 |
| D-Glucose-1-phosphate | 7^c^ | 2^c^ | 9^c^ |
| D-Fructose-6-phosphate | 17^c^ | 29 ^c^ | 52^c^ |
| Tween 80 | 0 | 0 | 1 |
| α-Hydroxyglutaric acid-γ-lactone | 1^c^ | 1^c^ | 1 |
| α-Hydroxybutyric acid | 10^c^ | 6^c^ | 15^c^ |
| β-Methyl-D-glucoside | 2 | 1^c^ | 6 |
| Adonitol | 3^c^ | 3^c^ | 8 |
| Maltotriose | 17^c^ | 32^c^ | 52^c^ |
| 2`-Deoxyadenosine | 7^c^ | 10^c^ | 15^c^ |
| Adenosine | 16^c^ | 18^c^ | 37^c^ |
| Gly-Asp | 12^c^ | 12^c^ | 15^c^ |
| Citric acid | 1^c^ | 0 | 2 |
| m-Inositol | 1^c^ | 0 | 2 |
| D-Threonine | 0 | 0 | 1 |
| Fumaric acid | 0^c^ | 1^c^ | 2^c^ |
| Bromosuccinic acid | 0 | 0 | 2 |
| Propionic acid | 4^c^ | 1^c^ | 2^c^ |
| Mucic acid | 2^c^ | 0^c^ | 2 |
| Glycolic acid | 0 | 0 | 2 |
| Glyoxylic acid | 0 | 0 | 1 |
| D-Cellobiose | 11^c^ | 10^c^ | 40^c^ |
| Inosine | 1 | 4 | 4^c^ |
| Gly-Glu | 3^c^ | 6^c^ | 10^c^ |
| Tricarballylic acid | 0 | 1 | 2 |
| L-Serine | 1^c^ | 1 | 1^c^ |
| L-Threonine | 3^c^ | 1 | 2^c^ |
| L-Alanine | 1^c^ | 0^c^ | 1 |
| Ala-Gly | 0 | 1^c^ | 1 |
| Acetoacetic acid | 2^c^ | 1^c^ | 4^c^ |
| N-Acetyl-D-mannosamine | 13^c^ | 27^c^ | 49^c^ |
| Mono-methylsuccinate | 2^c^ | 1 | 2 |
| Methylpyruvate | 13^c^ | 11^c^ | 32^c^ |
| D-Malic acid | 1 | 0 | 1 |
| L-Malic acid | 1^c^ | 0^c^ | 2^c^ |
| Gly-Pro | 2 | 2^c^ | 2 |
| p-Hydroxyphenyl acetic acid | 0^c^ | 0 | 1 |
| m-Hydroxyphenyl acetic acid | 1^c^ | 1 | 1 |
| Tyramine | 0^c^ | 0^c^ | 2 |
| D-Psicose | 17^c^ | 32^c^ | 54^c^ |
| L-Lyxose^b^ | 17^c^ | 32^c^ | 54^c^ |
| Glucuronamide | 17^c^ | 31^c^ | 54^c^ |
| Pyruvic acid | 16^c^ | 22^c^ | 48^c^ |
| L-Galactonic acid-γ-lactone | 0 | 0 | 2 |
| D-Galacturonic acid | 11^c^ | 26^c^ | 48^c^ |
| Phenylethylamine | 0^c^ | 0^c^ | 3 |
| 2-Aminoethanol | 1 | 0 | 2 |

^a^C = cow, G = goat, H = human and P = pig.

^b^Abiotic reaction.

^c^Type strain of the *Bacteroides* species used substrate.

| Supplemental Table 3. List of phenotypic microarray substrates and the number of isolates by *Bacteroides* species that utilized the substrates of Biolog PM2A. | | | |
| --- | --- | --- | --- |
| **Carbon substrate** | ***B. ovatus*** | ***B. thetaiotaomicron*** | ***B. xylanisolvens*** |
|  | n = 17 | n = 29 | n = 57 |
|  | 3 C, 14 H^a^ | 8 C, 5 G, 12 H, 7 P | 10 C, 26 G, 3 H, 15 P |
| Chondroitin sulfate C | 9^c^ | 26^c^ | 35^c^ |
| α-Cyclodextrin | 9^c^ | 21^c^ | 26^c^ |
| β-Cyclodextrin | 10^c^ | 28^c^ | 37^c^ |
| γ-Cyclodextrin | 10^c^ | 26^c^ | 43^c^ |
| Dextrin | 12^c^ | 27^c^ | 48^c^ |
| Gelatin | 5^c^ | 6^c^ | 10^c^ |
| Glycogen | 10^c^ | 22^c^ | 36^c^ |
| Inulin | 8^c^ | 3^c^ | 15^c^ |
| Laminarin | 6^c^ | 5^c^ | 16^c^ |
| Mannan | 7^c^ | 15^c^ | 32^c^ |
| Pectin | 13^c^ | 24^c^ | 47^c^ |
| N-Acetyl-D-galactosamine | 17^c^ | 30^c^ | 50^c^ |
| N-Acetyl-neuraminic acid | 0^c^ | 0 | 1^c^ |
| β-D-Allose | 17^c^ | 32^c^ | 54^c^ |
| Amygdalin | 9^c^ | 5^c^ | 37^c^ |
| D-Arabinose^b^ | 17^c^ | 32^c^ | 54^c^ |
| D-Arabitol | 2^c^ | 0 | 1 |
| L-Arabitol | 0 | 0 | 0 |
| Arbutin | 0^c^ | 3^c^ | 3^c^ |
| 2-Deoxy-D-ribose^b^ | 17^c^ | 32^c^ | 54^c^ |
| i-Erythritol | 0 | 0 | 3 |
| D-Fucose | 3^c^ | 11^c^ | 21^c^ |
| 3-O-β-D-Galactopyranosyl-D-arabinose | 17^c^ | 29^c^ | 51^c^ |
| Gentiobiose | 14^c^ | 25^c^ | 52^c^ |
| L-Glucose | 4^c^ | 10^c^ | 15^c^ |
| D-Lactitol | 8^c^ | 4^c^ | 28^c^ |
| D-Melezitose | 2^c^ | 2^c^ | 11^c^ |
| Maltitol | 6^c^ | 4^c^ | 28^c^ |
| α-Methyl-D-glucoside | 2^c^ | 1^c^ | 4^c^ |
| β-Methyl-D-galactoside | 17^c^ | 18^c^ | 44^c^ |
| 3-Methylglucose | 17^c^ | 32^c^ | 54^c^ |
| β-Methyl-D-glucuronic acid | 0^c^ | 6^c^ | 5^c^ |
| α-Methyl-D-mannoside | 4^c^ | 13^c^ | 19^c^ |
| β-Methyl-D-xyloside | 5^c^ | 14^c^ | 18^c^ |
| Palatinose^b^ | 17^c^ | 32^c^ | 53^c^ |
| D-Raffinose | 13^c^ | 20^c^ | 41^c^ |
| Salicin | 2 | 1 | 10 |
| Sedoheptulosan | 1^c^ | 2^c^ | 1 |
| L-Sorbose | 11^c^ | 23^c^ | 45^c^ |
| Stachyose | 9^c^ | 9^c^ | 9^c^ |
| D-Tagatose^b^ | 17^c^ | 32^c^ | 54^c^ |
| Turanose | 11^c^ | 25^c^ | 46^c^ |
| Xylitol | 0 | 1 | 2 |
| N-Acetyl-D-glucosaminitol | 0^c^ | 4 | 4 |
| γ-Amino-N-butyric acid | 0 | 4 | 3 |
| d-Amino valeric acid | 2 | 8^c^ | 7 |
| Butyric acid | 2^c^ | 8^c^ | 5 |
| Capric acid | 0 | 0 | 0 |
| Caproic acid | 0 | 0 | 1 |
| Citraconic acid | 0 | 0 | 0 |
| Citramalic acid | 0 | 0 | 1 |
| D-Glucosamine^b^ | 17^c^ | 32^c^ | 53^c^ |
| 2-Hydroxybenzoic acid^b^ | 0 | 1 | 1 |
| 4-Hydroxybenzoic acid | 0 | 1 | 2 |
| β-Hydroxybutyric acid | 1 | 8 | 7 |
| γ-Hydroxybutyric acid | 2 | 11^c^ | 17 |
| α-Keto-valeric acid | 5^c^ | 18^c^ | 27^c^ |
| Itaconic acid | 5 | 18^c^ | 27^c^ |
| 5-Keto-D-gluconic acid^b^ | 17^c^ | 32^c^ | 54^c^ |
| D-Lactic acid methyl ester | 1 | 1 | 2^c^ |
| Malonic acid | 0 | 0 | 1 |
| Melibionic acid | 3^c^ | 3^c^ | 9^c^ |
| Oxalic acid | 0 | 0 | 0 |
| Oxalomalic acid | 2^c^ | 3^c^ | 3^c^ |
| Quinic acid | 0^c^ | 0 | 1 |
| D-Ribono-1,4-lactone | 0 | 0 | 2 |
| Sebacic acid | 0 | 0 | 1 |
| Sorbic acid^b^ | 15^c^ | 15^c^ | 25^c^ |
| Succinamic acid | 0 | 2 | 2 |
| D-Tartaric acid | 1 | 1 | 1 |
| L-Tartaric acid | 0 | 1 | 1 |
| Acetamide | 0 | 0 | 2 |
| L-Alaninamide | 0 | 0 | 2 |
| N-Acetyl-L-glutamic acid | 0^c^ | 0 | 0 |
| L-Arginine | 0^c^ | 0 | 2 |
| Glycine | 0 | 0 | 0 |
| L-Histidine | 0 | 0 | 0 |
| L-Homoserine | 1 | 0 | 1 |
| Hydroxy-L-proline | 0^c^ | 1 | 2 |
| L-Isoleucine | 1^c^ | 1^c^ | 4 |
| L-Leucine | 1^c^ | 3^c^ | 7 |
| L-Lysine | 0^c^ | 3 | 3 |
| L-Methionine | 1^c^ | 2 | 3 |
| L-Ornithine | 0^c^ | 1 | 2^c^ |
| L-Phenylalanine | 0^c^ | 2^c^ | 2 |
| L-Pyroglutamic acid | 0^c^ | 0 | 1 |
| L-Valine | 0^c^ | 1^c^ | 1^c^ |
| D,L-Carnitine | 0^c^ | 0 | 2 |
| sec-Butylamine | 0 | 0 | 2 |
| D,L-Octopamine | 0^c^ | 2^c^ | 2 |
| Putrescine | 1 | 0 | 1 |
| Dihydroxyacetone^b^ | 17 | 32^c^ | 53^c^ |
| 2,3-Butanediol | 0^c^ | 1 | 1 |
| 2,3-Butanedione | 0 | 0 | 0 |
| 3-Hydroxy-2-butanone | 0 | 1 | 1 |

^a^C = cow, G = goat, H = human and P = pig.

^b^Abiotic reaction.

^c^Type strain of the *Bacteroides* species used substrate.

| Supplemental Table 4. List of phenotypic microarray substrates and the number of isolates by *Bacteroides* species that utilized the substrates of Biolog PM3B. | | | |
| --- | --- | --- | --- |
| **Nitrogen substrate** | ***B. ovatus*** | ***B. thetaiotaomicron*** | ***B. xylanisolvens*** |
|  | n = 17 | n = 32 | n = 54 |
|  | 3 C, 14 H^a^ | 8 C, 5 G, 12 H, 7 P | 10 C, 26 G, 3 H, 15 P |
| Ammonia | 0 | 0 | 3 |
| Nitrite | 0 | 0 | 2 |
| Nitrate | 0 | 0 | 0 |
| Urea | 0 | 0 | 0 |
| Biuret | 0 | 1 | 1 |
| L-Alanine | 0 | 0 | 1 |
| L-Arginine | 0 | 0 | 0 |
| L-Asparagine | 0 | 0 | 0 |
| L-Aspartic acid | 0 | 0 | 1 |
| L-Cysteine | 0 | 1 | 1 |
| L-Glutamic acid | 0 | 0 | 0 |
| L-Glutamine | 0 | 0 | 1 |
| Glycine | 0 | 1 | 0 |
| L-Histidine | 0 | 0 | 0 |
| L-Isoleucine | 0 | 0 | 0 |
| L-Leucine | 0 | 3 | 1 |
| L-Lysine | 0 | 1 | 1 |
| L-Methionine | 0 | 1 | 0 |
| L-Phenylalanine | 0 | 0 | 1 |
| L-Proline | 0 | 1 | 0 |
| L-Serine | 1 | 2 | 1 |
| L-Threonine | 0^b^ | 2 | 6 |
| L-Tryptophan | 0^b^ | 1 | 2 |
| L-Tyrosine | 0 | 1 | 1 |
| L-Valine | 0 | 0 | 0 |
| D-Alanine | 0 | 1 | 0 |
| D-Asparagine | 0 | 1 | 1 |
| D-Aspartic acid | 0 | 0 | 0 |
| D-Glutamic acid | 0 | 2 | 2 |
| D-Lysine | 0 | 1 | 2 |
| D-Serine | 0 | 6 | 4 |
| D-Valine | 1^b^ | 5^b^ | 5 |
| L-Citrulline | 2^b^ | 7^b^ | 13 |
| L-Homoserine | 4^b^ | 10^b^ | 13 |
| L-Ornithine | 4^b^ | 10^b^ | 15 |
| N-Acetyl-L-glutamic acid | 0 | 0 | 2 |
| N-Phthaloyl-L-glutamic acid | 0 | 0 | 1 |
| L-Pyroglutamic acid | 0 | 0 | 0 |
| Hydroxylamine | 0 | 0 | 0 |
| Methylamine | 0 | 1 | 0 |
| N-Amylamine | 0 | 1 | 0 |
| N-Butylamine | 0 | 1 | 3 |
| Ethylamine | 0^b^ | 2 | 1 |
| Ethanolamine | 0^b^ | 4 | 5 |
| Ethylenediamine | 2^b^ | 5 | 5 |
| Putrescine | 1^b^ | 7 | 7 |
| Agmatine | 1^b^ | 5^b^ | 5 |
| Histamine | 0 | 0 | 1 |
| b-Phenylethylamine | 0 | 0 | 1 |
| Tyramine | 0 | 0 | 1 |
| Acetamide | 0 | 0 | 2 |
| Formamide | 0 | 2 | 4 |
| Glucuronamide | 2 | 3 | 4 |
| DL-Lactamide | 3^b^ | 9^b^ | 9 |
| D-Glucosamine | 1^b^ | 9 | 15 |
| D-Galactosamine | 5^b^ | 13^b^ | 16 |
| D-Mannosamine | 6^b^ | 16^b^ | 28 |
| N-Acetyl-D-glucosamine | 5^b^ | 18^b^ | 25^b^ |
| N-Acetyl-D-galactosamine | 5^b^ | 16^b^ | 26^b^ |
| N-Acetyl-D-mannosamine | 0 | 1 | 1 |
| Adenine | 0 | 1 | 0 |
| Adenosine | 0 | 0 | 1 |
| Cytidine | 0 | 0 | 0 |
| Cytosine | 0 | 0 | 0 |
| Guanine | 17 | 31 | 50 |
| Guanosine | 0 | 0 | 0 |
| Thymine | 0 | 0 | 1 |
| Thymidine | 0 | 3 | 1 |
| Uracil | 0^b^ | 1 | 1 |
| Uridine | 0^b^ | 1 | 2 |
| Inosine | 0^b^ | 1 | 1 |
| Xanthine | 17^b^ | 32^b^ | 53^b^ |
| Xanthosine | 0 | 0 | 2 |
| Uric acid | 2^b^ | 4^b^ | 6^b^ |
| Alloxan | 0 | 0 | 1 |
| Allantoin | 1 | 0 | 0 |
| Parabanic acid | 1 | 0 | 1 |
| DL-α-Amino-N-butyric acid | 1 | 1 | 0 |
| γ-Amino-N-butyric acid | 0 | 0 | 1 |
| ε-Amino-N-caproic acid | 1 | 1 | 1 |
| DL-α-Amino-caprylic acid | 3^b^ | 5^b^ | 13 |
| d-Amino-N-valeric acid | 0^b^ | 2 | 1 |
| α-Amino-N-valeric acid | 0^b^ | 3 | 1 |
| Ala-Asp | 0 | 0 | 1 |
| Ala-Gln | 0 | 0 | 0 |
| Ala-Glu | 0 | 0 | 0 |
| Ala-Gly | 0 | 0 | 1 |
| Ala-His | 0 | 0 | 3 |
| Ala-Leu | 0 | 0 | 2 |
| Ala-Thr | 0 | 0 | 2 |
| Gly-Asn | 0 | 1 | 2 |
| Gly-Gln | 0 | 0 | 1 |
| Gly-Glu | 0 | 0 | 3 |
| Gly-Met | 0 | 0 | 1 |
| Met-Ala | 0 | 0 | 0 |

^a^C = cow, G = goat, H = human and P = pig.

^b^Type strain of the *Bacteroides* species used substrate.
